# Supplementary material for: A “Population Dynamics” Perspective on the Delayed Life-History Effects of Environmental Contaminations: An Illustration with a Preliminary Study of Cadmium Transgenerational Effects over Three Generations in the Crustacean Gammarus
Source: Int J Mol Sci. 2020 Jul 1;21(13):4704. doi: 10.3390/ijms21134704 (PMC7370439; doi:10.3390/ijms21134704)
Supplement: Supplementary file 1 [file ijms-21-04704-s001.pdf]

Supporting Information

A "population dynamics" perspective on the delayed life-history effects of environmental contaminations: an illustration with a preliminary study of cadmium transgenerational effects over three generations in the crustacean *Gammarus*

Pauline Cribiu [1,2], Alain Devaux [2], Laura Garnero [1], Khédidja Abbaci [1], Thérèse Bastide [2], Nicolas Delorme [1], Hervé Quéau [1], Davide Degli Esposti [1], Jean-Luc Ravanat [3], Olivier Geffard [1], Sylvie Bony [2], Arnaud Chaumot [1,*]

[1] INRAE, UR RiverLy, Laboratoire d'écotoxicologie, 5 rue de la Doua CS 20244, F-69625 Villeurbanne, France
[2] ENTPE, INRAE, CNRS UMR 5023 LEHNA, rue Maurice Audin 69518 Vaulx-en-Velin CEDEX, France
[3] CEA, LAN, 17 rue des martyrs 38054 Grenoble CEDEX 9, France
* Correspondence: arnaud.chaumot@inrae.fr (A.C.)

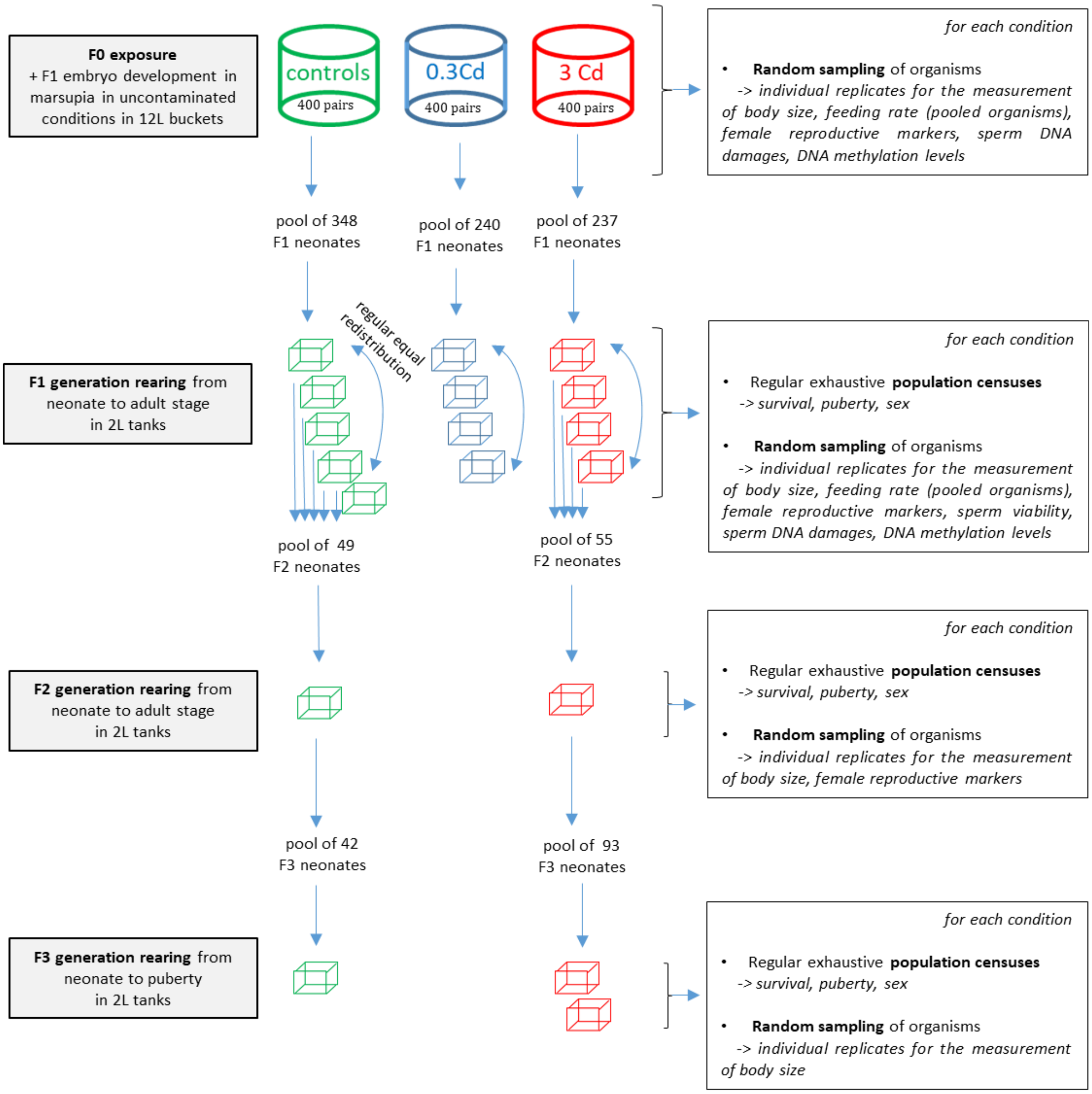

Figure S1: Description of experimental design and the schedule for the measurement of biological endpoints over the generations

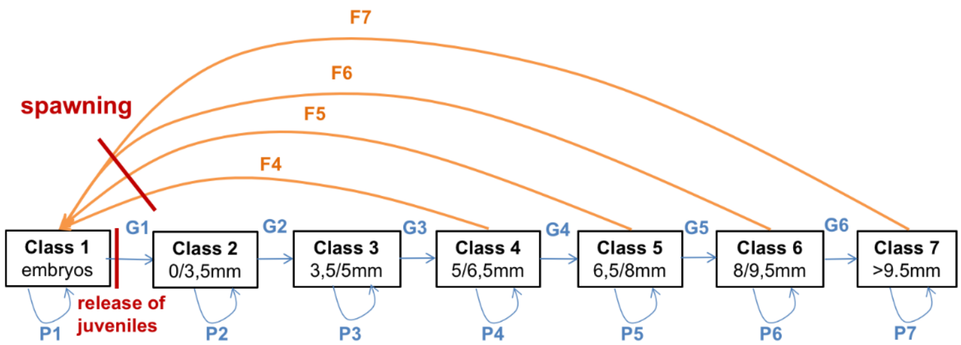

Figure S2: Life cycle graph of the *Gammarus fossarum* laboratory population with 7 size-classes. P_i = proportion of surviving and remaining organisms in size-class *i*, G_i = proportion of surviving and moving individuals from size-class *i* to size-class *i*+1, F_i = fertility in size-class *i*.

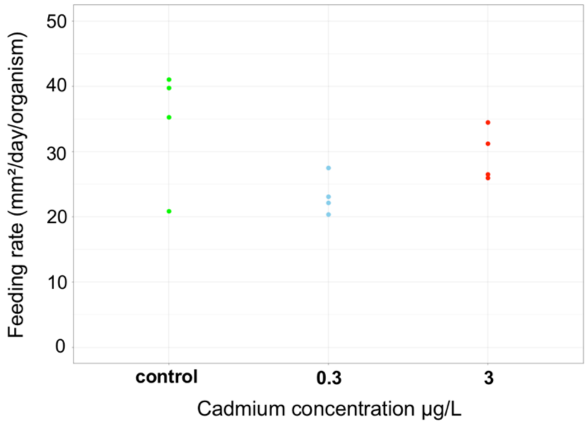

Figure S3: Feeding rate of F0 parents. n = 4.

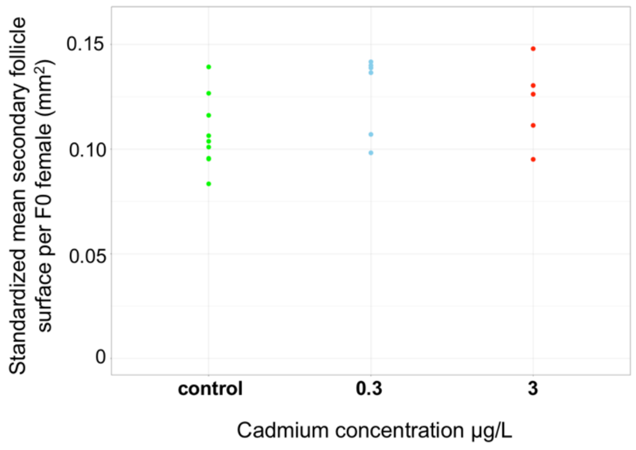

Figure S4: Standardized mean secondary follicle surface of F0 females. n = 9 (control), n = 6 (0.3 µg Cd /L) and n = 5 (3 Cd µg/L).

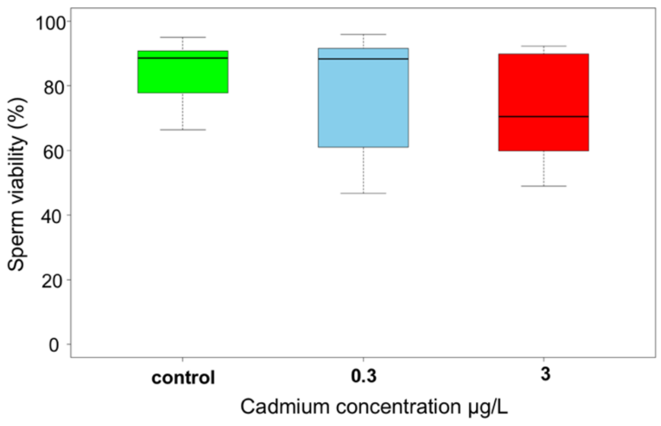

Figure S5: Sperm viability in F0 male gammarids. Control: n = 10, 0.3 µg Cd/L: n=9, 3 µg Cd /L: n=8.

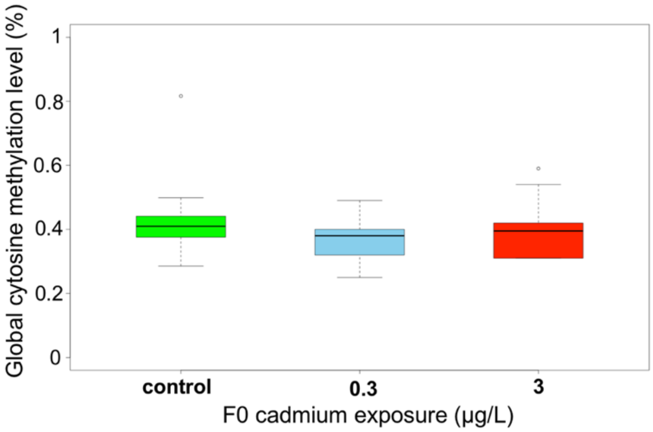

Figure S6: Global cytosine methylation level in F0 male gammarids. Control: n = 10, 0.3 µg Cd/L: n=11, 3 µg Cd /L: n=10.

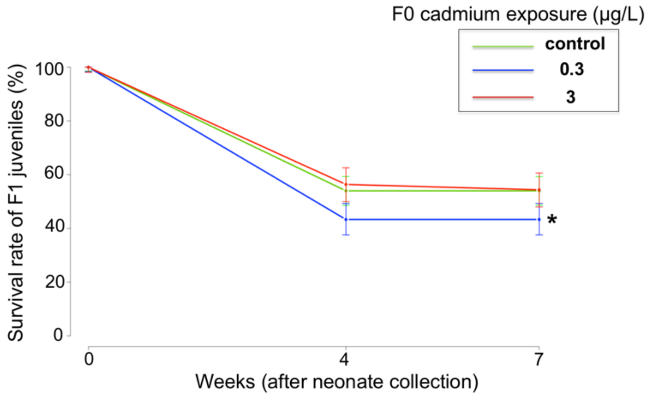

Figure S7: Survival of F1 juveniles. The star denotes a significant difference between C-F0 and 0.3Cd-F0 conditions, $p<0.05$.

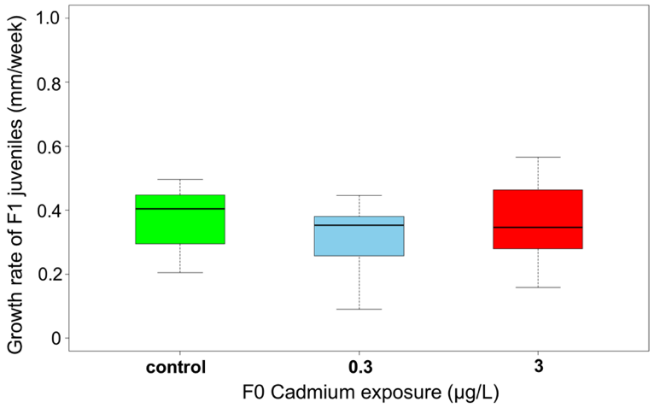

Figure S8: Weekly growth rate of F1 juveniles. n=20.

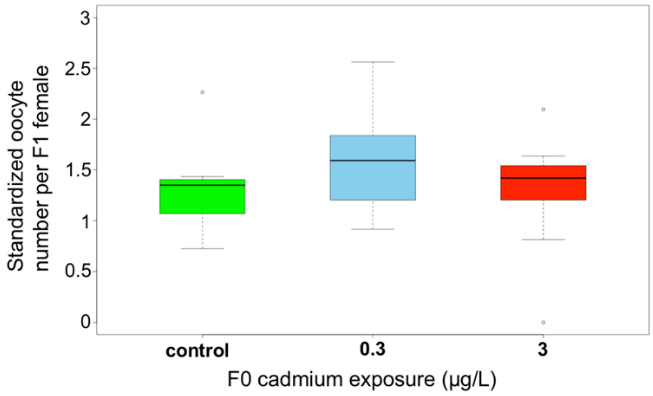

Figure S9: Oocyte production in F1 generation (size-standardized oocyte number per females). n=10.

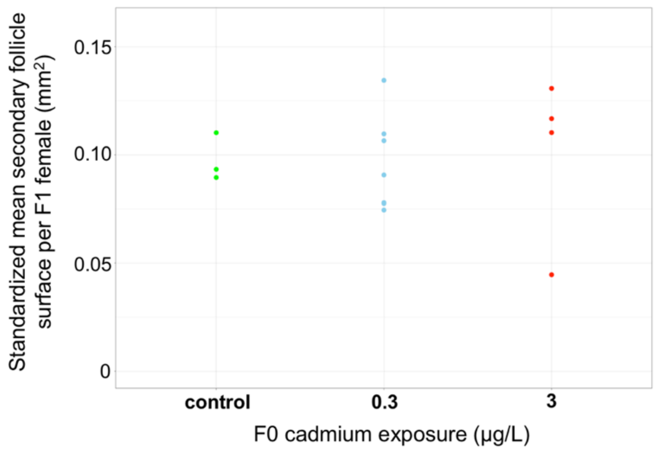

Figure S10: Standardized mean secondary follicle surface of F1 females. Control: n = 3, 0.3 µg Cd/L: n=7, 3 µg Cd/L: n=4.

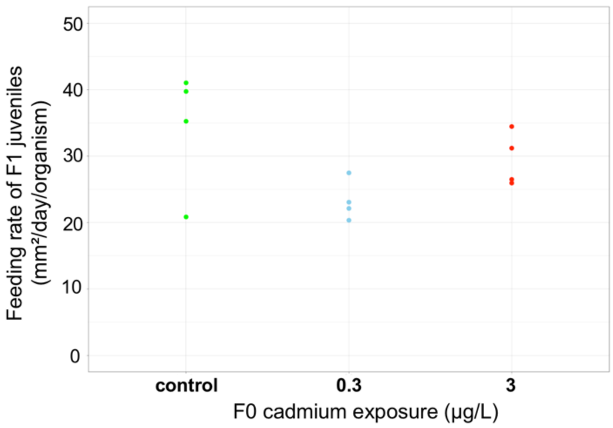

Figure S11: Feeding rate of F1 males. n = 4.

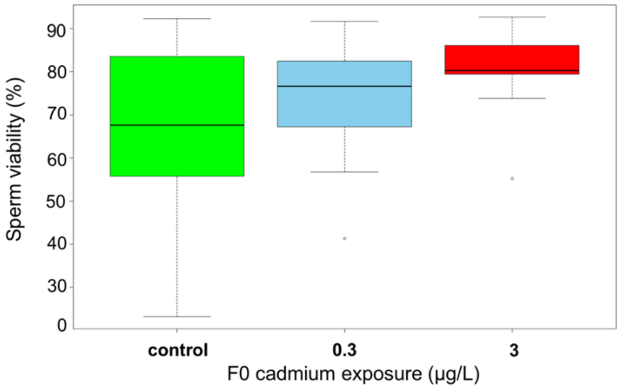

Figure S12: Sperm viability in F1 males. Control: n = 9, 0.3 µg Cd/L: n=9, 3 µg Cd/L: n=10.

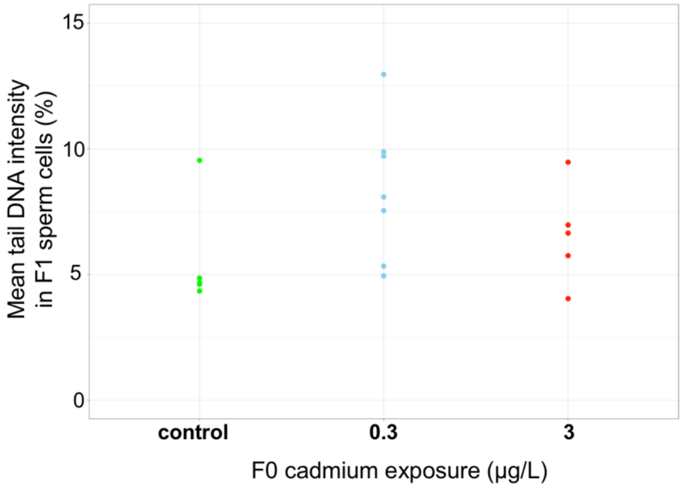

Figure S13: Mean tail DNA intensity (comet assay) in sperm of F1 males. Control: n = 5, 0.3 µg Cd/L: n=7, 3 µg Cd/L: n=5.

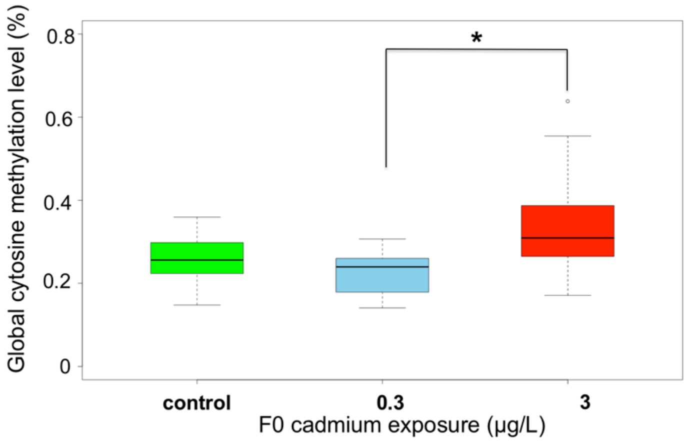

Figure S14: Global cytosine methylation level in F1 males. Control: n = 10, 0.3 µg Cd/L: n=9, 3 µg Cd /L: n=14. The star denotes a significant difference between 0.3Cd-F0 and 3Cd-F0 conditions, $p<0.05$.

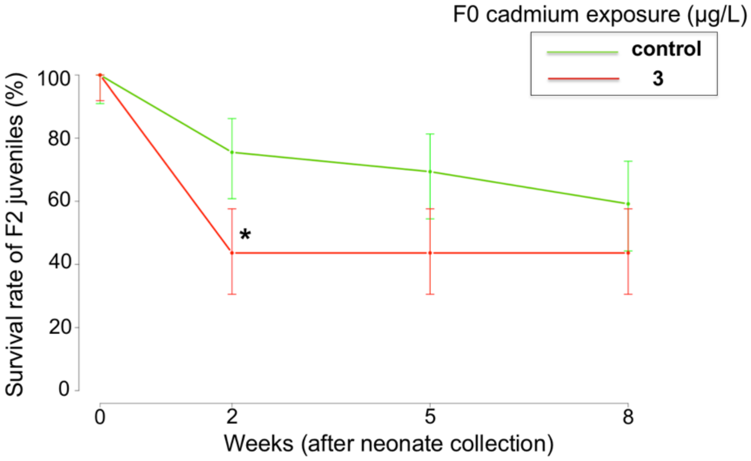

Figure S15: Survival of F2 juveniles. The star denotes a significant difference between conditions.

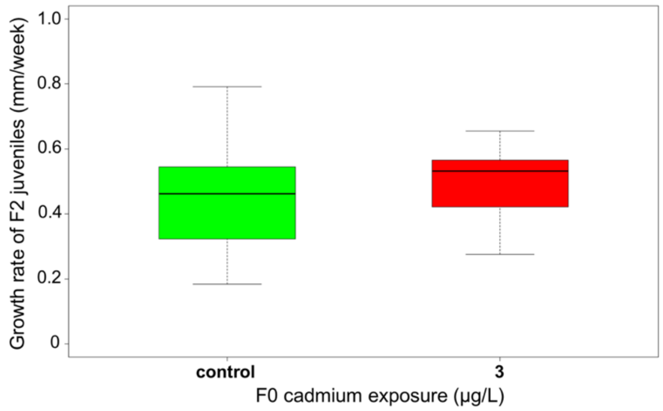

Figure S16: Weekly growth rate of F2 juveniles. Control: n = 15, 3 µg Cd/L: n=13.

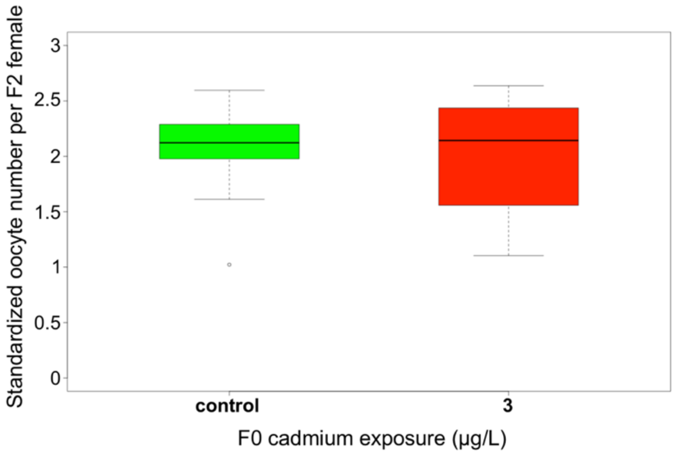

Figure S17: Oocyte production in F2 generation (size-standardized oocyte number per females). Control: n = 11, 3 µg Cd/L: n=10.

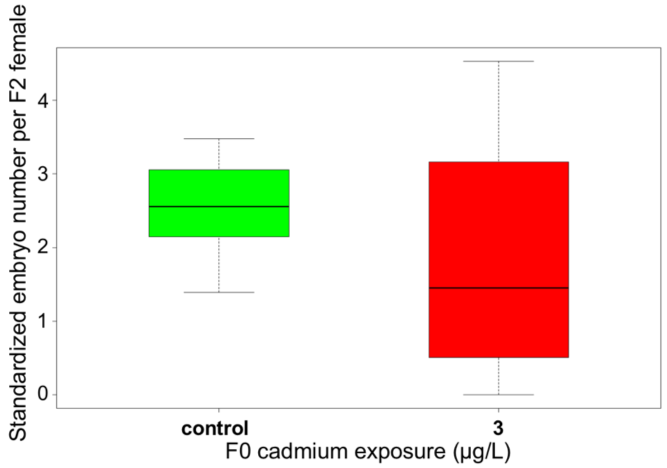

Figure S18: Embryo number per female in F2 generation (size-standardized embryo number). Control: n = 11, 3 µg Cd/L: n=7.

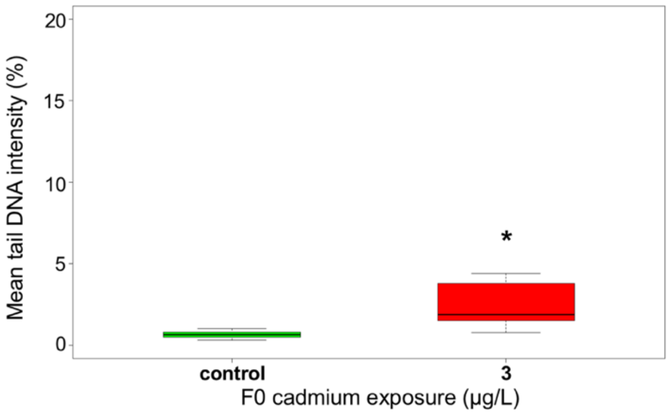

Figure S19: Mean tail DNA intensity (comet assay) in sperm of F2 males. Control: n = 7, 3 µg Cd/L: n=7. The star denotes a significant difference between conditions, $p<0.05$.
